# P-1090. Post-discharge Decolonization of Methicillin Resistant Staphylococcus aureus carriers: A Survey of Current Practices in U.S. Hospitals

**DOI:** 10.1093/ofid/ofaf695.1285

**Published:** 2026-01-11

**Authors:** Nasia Safdar, Julie Keating, Linda McKinley, Marin Schweizer

**Affiliations:** University of Wisconsin School of Medicine and Public Health, Madison, WI; Madison VA Hospital, Madison, Wisconsin; Wm. S. Middleton Memorial VA Hospital, Madison, Wisconsin; William S. Middleton VA Hospital, madison, Wisconsin

## Abstract

**Background:**

Universal or targeted Methicillin Resistant *Staphylococcus aureus* (MRSA) screening and isolation is a common pathogen-specific infection control and prevention strategy in hospitalized patients. MRSA carriers are at high risk for subsequent MRSA infection and decolonization strategies have been used to reduce this risk. Decolonization of MRSA carriers often occurs in high-risk hospitalized patient populations such as prior to invasive procedures (i.e., surgery), those who have invasive devices (i.e., central lines) or for those who are critically ill. However, little guidance is available for MRSA carriers following hospital discharge in those who have not received decolonization treatment. The survey aimed to better understand the current practices of post-discharge MRSA decolonization prevention strategies within U.S. healthcare facilities.

**Methods:**

We conducted a cross-sectional electronic survey of hospital epidemiologists across Infectious Disease Society of America Emerging Infections Network, a provider-based emerging infections sentinel network. The 6-question survey was developed by a team including health services researchers, infectious disease physicians, and infection preventionists.

**Results:**

A total of 185 individuals responded to the survey between March 25 and April 9, 2025. Eleven percent (21 of 185) of respondents indicated they routinely performed post-discharge MRSA decolonization. The top three patient populations targeted for post-discharge decolonization were: 1) patients identified as MRSA carriers (colonization or infection) during hospitalization (62%), 2) MRSA carriers with a recent history, within 90-days, of MRSA infection or colonization (30%), and 3) MRSA carriers with invasive lines (19%). All respondents (100%) reported using topical skin antiseptics for decolonization and most (86%) used topical nasal antibiotic (mupirocin).

**Conclusion:**

Post-discharge MRSA decolonization practices were reportedly low among survey respondents despite recent study finding it significantly lowers infection risk. Healthcare organizations and professional guidance are needed to facilitate initiatives to address this gap.

**Disclosures:**

Linda McKinley, RN, PhD, MPH, CIC, FAPIC, Molnlycke: Advisor/Consultant

